# Risk factors for prevalent and incident hypertension in rheumatoid arthritis: data from the Canadian Early Arthritis Cohort

**DOI:** 10.1093/rap/rkae066

**Published:** 2024-05-22

**Authors:** Brook Hadwen, Saverio Stranges, Janet E Pope, Susan Bartlett, Gilles Boire, Louis Bessette, Carol A Hitchon, Glen Hazlewood, Edward C Keystone, Orit Schieir, Carter Thorne, Diane Tin, Marie-France Valois, Vivian Bykerk, Lillian Barra, CATCH Investigators, Pooneh Akhavan, Louis Bessette, Gilles Boire, Vivian Bykerk, Ines Colmegna, Sabrina Fallavollita, Derek Haaland, Boulos Haraoui, Glen Hazlewood, Carol Hitchon, Shahin Jamal, Raman Joshi, Ed Keystone, Bindee Kuriya, Peter Panopalis, Janet Pope, Carter Thorne, Edith Villeneuve, Michel Zummer

**Affiliations:** Department of Epidemiology and Biostatistics, University of Western Ontario, London, Ontario, Canada; Department of Epidemiology and Biostatistics, University of Western Ontario, London, Ontario, Canada; Department of Family Medicine, Western University, London, Ontario, Canada; Lawson Health Research Institute, London, Ontario, Canada; Department of Population Health, Luxembourg Institute of Health, Strassen, Luxembourg; Department of Medicine, Division of Rheumatology, Western University, London, Ontario, Canada; Department of Medicine, Division of Rheumatology, McGill University, Montreal, Quebec, Canada; Department of Medicine, Division of Rheumatology, Centre Intégré Universitaire de Santé et de Services Sociaux de l’Estrie—Centre Hospitalier Universitaire de Sherbrooke and Université de Sherbrooke, Sherbrooke, Quebec, Canada; Department of Medicine, Division of Rheumatology, Université Laval, Quebec, Quebec, Canada; Department of Internal Medicine, Section of Rheumatology, University of Manitoba, Winnipeg, Manitoba, Canada; Department of Medicine, Division of Rheumatology, University of Calgary, Calgary, Alberta, Canada; Department of Medicine, Division of Rheumatology, University of Toronto, Toronto, Ontario, Canada; Department of Medicine, Division of Rheumatology, McGill University, Montreal, Quebec, Canada; Centre of Arthritis Excellence, Newmarket, Ontario, Canada; Centre of Arthritis Excellence, Newmarket, Ontario, Canada; Department of Medicine, Division of Rheumatology, McGill University, Montreal, Quebec, Canada; Department of Medicine, Hospital for Special Surgery, Weill Cornell Medical, New York, NY, USA; Department of Epidemiology and Biostatistics, University of Western Ontario, London, Ontario, Canada; Lawson Health Research Institute, London, Ontario, Canada; Department of Medicine, Division of Rheumatology, Western University, London, Ontario, Canada

**Keywords:** rheumatoid arthritis, hypertension, comorbidities, cardiovascular disease, cohort

## Abstract

**Objective:**

Hypertension (HTN) is a common comorbidity in RA. This study aimed to explore the prevalence and incidence of HTN and baseline factors associated with incident HTN in early RA (ERA).

**Methods:**

Data were from the Canadian Early Arthritis Cohort (CATCH), an inception cohort of ERA patients having <1 year of disease duration. HTN was determined by patient- or physician-reported diagnosis, the use of anti-hypertensives and/or blood pressure. Multivariable logistic regression was performed to determine baseline factors associated with prevalent and incident HTN in this population.

**Results:**

The study sample included 2052 ERA patients [mean age 55 years (s.d. 14), 71% female). The prevalence of HTN at study enrolment was 26% (23% in females and 34% in males). In both sexes, prevalent HTN was associated with older age, diabetes and hyperlipidaemia. HTN was associated with being overweight or high alcohol consumption in females. Of the RA patients who did not have HTN at enrolment, 24% (364/1518) developed HTN during the median follow-up period of 5 years (range 1–8). Baseline factors significantly associated with incident HTN were older age, being overweight, excess alcohol consumption and having hyperlipidaemia. Incident HTN was associated with high alcohol consumption in males and with hyperlipidaemia in females. RA-associated disease factors and treatments were not significantly associated with prevalent or incident HTN.

**Conclusions:**

Early RA patients had a high incidence of hypertension with the highest risk in older patients with traditional cardiovascular risk factors.

## Graphical Abstract

**Graphical Abstract:**
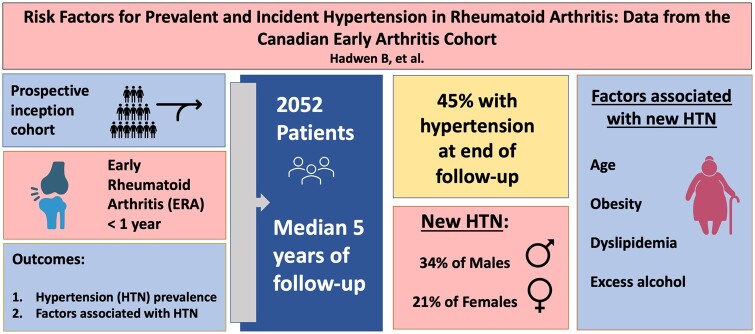

Key messages

- Hypertension is common in early RA, affecting 40% after 5 years of follow-up.
- Male RA patients were more likely to have hypertension.
- Older patients and those with traditional cardiovascular risk factors were at highest hypertension risk.

## Introduction

RA is characterized by joint swelling and systemic inflammation leading to pain, functional impairment and an increased risk of comorbidities. At diagnosis, most RA patients (75%) have at least one comorbidity [1]. Patients with comorbidities are less likely to achieve remission and more likely to have impaired physical function [2], emphasizing the necessity of preventing and treating comorbidities early in the disease course.

One of the most common RA comorbidities is hypertension (HTN), reported to occur in up to 80% of patients with long-term disease and more frequent in males [1, 3, 4]. In RA, HTN is the greatest risk factor for cardiovascular (CV) disease [5, 6], the leading cause of death in RA patients [3]. Compared with the general population, RA patients are 50–60% more likely to experience CV events and CV death [7, 8]. These events appear to occur later in the RA disease course [9, 10], thus there may be a window of opportunity to identify and modify CV risk factors in early RA and prevent future complications.

Unfortunately, there is a lack of studies investigating the incidence of HTN and factors associated with incident HTN in early RA patients. Prior studies of patients with established RA mostly investigated the relationship between medications and incident hypertension [11]. Cyclooxygenase-2 (COX2) inhibitors and glucocorticoids are known to increase the risk of incident HTN in many populations, including RA patients, while methotrexate was found to be protective in several studies [11]. The effects of other drugs and behavioural and RA-related factors on HTN remain unclear based on the available literature [11].

Given that the frequency of CV disease risk factors differs by sex [12], this study aimed to determine the prevalence and incidence of HTN in male and female patients with early RA and identify sex differences in baseline factors associated with HTN using a large Canadian prospective cohort of early RA patients.

## Methods

### Population

The Canadian Early Arthritis Cohort (CATCH) study is an ongoing multicentre inception cohort of early inflammatory arthritis patients (<12 months of persistent synovitis) established in 2007. Details regarding enrolment, inclusion criteria and data collection have been previously described [2, 6, 13]. On 24 March 2017, a more comprehensive data collection of CV disease risk factors and blood pressure (BP) measures at follow-up was initiated, thus the participants who had withdrawn from the study prior to 2017 were excluded from the analyses (Supplementary Fig. S1, available at *Rheumatology Advances in Practice* online). Study follow-up visits were every 3 months for the first year, at 18 months and then annually. At these appointments, patients underwent a history and physical exam where RA disease activity was determined by a rheumatologist, BP and body mass index (BMI) measurements were obtained and questionnaires were completed that included physician-diagnosed comorbidities and medication history. The sample for this study included 2052 patients enrolled between 2007 and 2020 with at least 1 year of follow-up and one visit after 2016. The ethics committees of all participating CATCH sites approved the study and written informed consent was obtained from all study participants.

### Measures

#### Outcome variables

There were two primary outcomes for this study: prevalence of HTN at study enrolment (baseline) and incident HTN. HTN at baseline was defined as either reported by the rheumatologist based on history, physical examination or whether the patient was taking anti-hypertensive medications or by an answer of yes to the following patient survey question: ‘Have you been diagnosed by a physician with high blood pressure?’. A binary incident HTN variable was created, indicating whether the patient developed new HTN at any time after baseline over the entire follow-up period (those who were hypertensive at baseline were excluded). In addition to the HTN definition that was used at baseline, a participant was deemed to have HTN over the follow-up period based on BP measurements recorded at study follow-up visits. The method for obtaining BP was not standardized across centres but generally was taken once in one arm after 5 min of rest in the sitting position. The following BP measurements were considered hypertensive according to Canadian Hypertension Guidelines: a measurement of systolic BP ≥180 mmHg or diastolic BP ≥110 mmHg once or BP measurements of systolic BP ≥140 mmHg or diastolic BP ≥90 mmHg at two consecutive visits over the follow-up [14].

#### Other variables

Demographics, behaviours, comorbidities, medications and RA clinical variables were collected at baseline. Sex, race, education and behaviours were self-reported. Tobacco smoking was defined as being a past, current or never smoker. Alcohol servings per week were dichotomized as exceeding or not exceeding Canada’s 2011 Low-Risk Alcohol Drinking Guidelines for males and females [15]. BMI was calculated according to patient-reported or physician-measured height and weight and categorized according to the World Health Organization standards [16]. Symptom duration was defined as when the patients first reported RA symptoms until the time of enrolment into the study. The 28-joint DAS (DAS28) was calculated using ESR or, if unavailable, CRP. Seropositivity was defined as being RF positive and/or ACPA positive and seronegative as being both RF and ACPA negative. All comorbidities were based on patient reports of physician-diagnosed conditions or reported by the rheumatologist based on clinical assessment or chart review. The CV disease variable was created as a composite of angina, myocardial infarction, coronary artery disease, coronary artery bypass graft surgery, coronary angioplasty, peripheral vascular disease and cerebral vascular accident. Medications included in the analyses were methotrexate, hydroxychloroquine, sulfasalazine, any oral glucocorticoid and any NSAID. Other RA therapies were not included in the analyses because the number of patients taking these drugs at baseline was low. NSAID use was defined as current use (i.e. use within 28 days of entry into the cohort). Other medication use was in the pre-enrolment period (365 to 28 days prior to entry into the cohort).

### Statistical analysis

All statistical analyses were conducted using Stata version 16 (StataCorp, College Station, TX, USA). Descriptive statistics were used to report mean (s.d.) or median [interquartile range (IQR)] for continuous variables and number (%) for categorical variables. Multivariable logistic regression models were built to identify baseline risk factors for prevalent HTN at study enrolment and incident HTN over the follow-up period. Variables were included in the multivariable logistic models based on prior knowledge of risk factors for HTN or the goodness of fit by the likelihood ratio. Multicollinearity was assessed by checking the variance inflation factor (VIF), where a VIF >5 suggested multicollinearity. Linearity of continuous independent variables with the log odds was assessed using the Box–Tidwell test. Analyses were subsequently stratified by sex. *P*-values were two-sided with a significance level of α = 0.05 considered significant. Subjects with missing data on any of the variables included in the multivariable logistic regression models were excluded from the model.

## Results

### Characteristics of the study population

There were 2052 ERA patients who met the eligibility criteria for the study. The characteristics of the study population at baseline can be found in Table 1. The majority were female and White with a mean age of 55 years (s.d. 14) and 68% were seropositive for RF and/or ACPA. Patients experienced a mean symptom duration of 5.60 months (s.d. 2.95) and the RA disease activity at baseline was moderate with a mean DAS28 score of 4.84 (s.d. 1.47). Almost half of the sample was taking NSAIDs at the time of enrolment, 28% were taking oral corticosteroids and 90% were taking at least one DMARD, with methotrexate being most common. Traditional CV risk factors were common: 56% had a history of tobacco smoking, 67% were overweight or obese, 20% had hyperlipidaemia and 11% had diabetes. There were several significant sex differences in baseline characteristics. Females were younger and more likely to have a post-secondary education, have elevated acute phase reactants, be seropositive for RF and/or ACPA, have comorbid depression and use NSAIDs. Males more commonly had traditional risk factors for CV disease (diabetes, hyperlipidaemia, smoking and obesity) and higher creatinine and were more likely to have excessive alcohol consumption (Table 1).

**Table 1.** Characteristics of the study population

Variable		Total (*N* = 2052)	Females (*n* = 1438)	Males (*n* = 590)	*P*-value	Missing, *n* (%)
Demographics						
Female, *n* (%)		1438 (71)				24 (1)
Age, years, mean (s.d.)		55.25 (14.23)	53.20 (14.32)	60.23 (12.69)	<0.0001	28 (1)
White, *n* (%)		1709 (85)	1186 (83)	523 (90)	<0.001	42 (2)
Post-secondary education, *n* (%)		1190 (61)	881 (63)	309 (54)	<0.001	92 (4)
Behaviours, *n* (%)						
Smoking	Never	879 (44)	694 (49)	181 (32)	<0.001	59 (3)
	Past	797 (40)	511 (36)	284 (50)		
	Current	317 (16)	210 (15)	107 (19)		
Exceeds recommended alcohol servings/week		87 (4)	48 (3)	74 (13)	<0.001	56 (3)
BMI, *n* (%)	Underweight	43 (2)	38 (3)	2 (0)	<0.001	248 (12)
	Normal	562 (231)	444 (35)	109 (21)		
	Overweight	643 (36)	402 (32)	234 (46)		
	Obese	556 (31)	386 (30)	386 (76)		
Clinical						
Symptom duration, years, mean (s.d.)		5.60 (2.95)	5.70 (2.96)	5.38 (2.91)	0.0216	55 (3)
HAQ score, mean (s.d.)		0.59 (0.56)	0.60 (0.55)	0.58 (0.57)	0.4884	63 (3)
DAS-28, mean (s.d.)		4.84 (1.47)	4.84 (1.44)	4.85 (1.53)	0.9376	141 (7)
RF positive, *n* (%)		1198 (60)	871 (62)	313 (54)	0.002	48 (2)
ACPA positive, *n* (%)		1047 (57)	757 (59)	276 (53)	0.029	225 (11)
RF and/or ACPA positive, *n* (%)		1369 (68)	995 (70)	357 (61)	<0.0001	24 (1)
ESR, mm/h, mean (s.d.)		26.63 (21.93)	27.36 (22.12)	24.89 (21.29)	0.031	256 (12)
CRP, mg/l, mean (s.d.)		14.82 (19.16)	13.57 (17.71)	18.15 (22.20)	<0.0001	178 (9)
Creatinine, µmol/l, mean (s.d.)		65 (19.0)	63.01 (13.04)	77.87 (16.20)	<0.0001	255 (12)
Creatinine >125 µmol/l, *n* (%)		9 (0)	2 (0)	7 (1)	0.003	255 (12)
Medication, *n* (%)						
NSAIDs	At baseline	877 (49)	662 (52)	213 (42)	<0.001	263 (13)
MTX	Pre-baseline	308 (15)	205 (14)	102 (17)	0.084	0 (0)
HCQ	Pre-baseline	377 (18)	262 (18)	115 (19)	0.504	0 (0)
SSZ	Pre-baseline	79 (4)	55 (4)	24 (4)	0.797	0 (0)
Other DMARD	Pre-baseline	15 (1)	10 (1)	5 (0)	0.93	0 (0)
Oral corticosteroids	Pre-baseline	274 (13)	129 (9)	60 (10)	0.399	0 (0)
Biologics or JAK inhibitors	Pre-baseline	4 (0)	2 (0)	2 (0)	0.585	0 (0)
Comorbidities, *n* (%)						
Hyperlipidaemia		378 (20)	193 (15)	182 (34)	<0.001	185 (9)
Diabetes		186 (11)	99 (8)	87 (17)	<0.001	315 (15)
Depression		212 (11)	178 (13)	32 (6)	<0.001	154 (8)

High alcohol intake was defined as >10 servings for females and >15 servings for males per week. BMI: normal, 18.5–24.9 kg/m^2^; overweight, 25.0–29.9 kg/m^2^; obese, ≥30.0 kg/m^2^).

MTX: methotrexate; HCQ: hydroxychloroquine; SSZ: sulfasalazine; JAK: Janus kinase.

### Prevalent hypertension at baseline

Within our study cohort, 537 (26%) subjects had a diagnosis of HTN at or prior to study enrolment. This was more common in males, with 210/614 (34%) being hypertensive compared with 327/1438 (23%) females. The following characteristics were significantly associated with HTN in multivariable regression analyses (Table 2): being ≥50 years of age [odds ratio (OR) 4.83 (95% CI 3.18, 7.34)], being overweight [OR 1.62 (95% CI 1.12, 2.33)] or obese [OR 2.75 (95% CI 1.91, 3.95)] and having diabetes [OR 3.52 (95% CI 2.29, 5.42)] or hyperlipidaemia [OR 2.49 (95% CI 1.79, 3.46)]. Smoking history and RA disease-related factors were not associated with prevalent HTN. For most variables, the association with HTN was not different between sexes. Excess weight was more strongly associated with HTN in females *vs* males. Similarly, exceeding recommended servings of alcohol was associated with being hypertensive in females [OR 2.91 (95% CI 1.15, 7.36)], but this association was not statistically significant in males. HAQ, baseline NSAID use or DMARD and corticosteroid use or dose in the pre-enrolment period were not included in the final model because they did not significantly improve the fit of the model.

**Table 2.** Multivariable logistic regression analyses of baseline associations with prevalent HTN

Variable	Adjusted OR (95% CI)		
	Total (*N* = 1334)	Females (*n* = 957)	Males (*n* = 377)
Demographics			
Age ≥50 years	4.83 (3.18, 7.34)	4.56 (2.83, 7.32)	5.82 (2.32, 14.61)
Female	1.01 (0.74, 1.37)		
White	0.91 (0.59, 1.40)	0.94 (0.57, 1.54)	0.75 (0.32, 1.79)
Post-secondary education	0.84 (0.64, 1.12)	0.76 (0.54, 1.08)	1.05 (0.64, 1.72)
Behaviours			
Current *vs* never smoker	0.71 (0.46, 1.10)	0.78 (0.46, 1.30)	0.61 (0.27, 1.37)
Past smoker *vs* never smoker	1.09 (0.80, 1.48)	0.99 (0.68, 1.45)	1.42 (0.80, 2.50)
High alcohol intake	1.33 (0.68, 2.61)	2.91 (1.15, 7.36)	0.52 (0.19, 1.45)
Clinical			
Overweight *vs* normal BMI	1.62 (1.12, 2.33)	1.83 (1.17, 2.85)	1.14 (0.59, 2.21)
Obese *vs* normal BMI	2.75 (1.91, 3.95)	2.98 (1.92, 4.62)	1.96 (0.99, 3.88)
Symptom duration, months	0.97 (0.93, 1.02)	0.96 (0.90, 1.02)	1.00 (0.92, 1.08)
DAS-28	1.01 (0.92, 1.12)	1.02 (0.90, 1.15)	1.03 (0.88, 1.20)
RF positive and/or ACPA positive	0.93 (0.70, 1.24)	1.00 (0.69, 1.44)	0.75 (0.46, 1.23)
Comorbidities			
Diabetes	3.52 (2.29, 5.42)	3.84 (2.12, 6.95)	3.11 (1.64, 5.90)
Hyperlipidaemia	2.49 (1.79, 3.46)	2.82 (1.82, 4.38)	2.02 (1.21, 3.36)
Depression	1.03 (0.65, 1.63)	1.01 (0.60, 1.69)	0.91 (0.29, 2.90)

BMI: overweight, 25.0–29.9 kg/m^2^; obese, ≥30.0 kg/m^2^.

High alcohol intake defined as >10 servings for females and >15 servings for males per week.

### Incident HTN

Of those who did not have HTN at baseline (*n* = 1515), 367 (24%) developed HTN over the study follow-up period at a median of 5 years (range 1–8). By sex, 21% of females and 34% of males developed incident HTN. Multivariable logistic regression was performed to identify baseline variables associated with incident HTN over the study follow-up period (Table 3). For the total sample, traditional CV risk factors such as older age [OR 2.46 (95% CI 1.73, 3.51)], being overweight [OR 1.95 (95% CI 1.33, 2.85)] or obese [OR 3.52 (95% CI 2.36, 5.24)] and having hyperlipidaemia [OR 1.69 (95% CI 1.07, 2.66)] were associated with incident HTN. Other significant factors were excess alcohol consumption [OR 2.20 (95% CI 1.04, 4.66)]. Incident HTN was not significantly associated with smoking or DAS28. Medications and HAQ were not added to the regression model because they did not improve model fit. The following factors were associated with incident HTN more significantly in females than males: White race, being overweight and having hyperlipidaemia. Conversely, excess alcohol had a higher risk of HTN in males.

**Table 3.** Multivariable logistic regression analyses of baseline associations with incident HTN

Variable	Adjusted OR (95% CI)		
	Total (*N* = 958)	Females (*n* = 718)	Males (*n* = 240)
Demographics			
Age ≥50 years	2.46 (1.73, 3.51)	3.04 (1.99, 4.64)	1.17 (0.58, 2.36)
Female	0.86 (0.60, 1.23)		
White	1.41 (0.86, 2.30)	1.91 (1.05, 3.44)	0.58 (0.20, 1.68)
Post-secondary education	1.41 (1.03, 1.96)	1.33 (0.90, 1.96)	1.54 (0.83, 2.86)
Behaviours			
Current smoker *vs* never smoker	1.02 (0.65, 1.59)	1.08 (0.63, 1.85)	0.65 (0.28, 1.53)
Past smoker *vs* never smoker	1.16 (0.82, 1.65)	1.15 (0.76, 1.73)	1.26 (0.61, 2.59)
High alcohol intake	2.20 (1.04, 4.66)	1.24 (0.34, 4.47)	4.39 (1.50, 12.87)
Clinical			
Overweight *vs* normal BMI	1.95 (1.33, 2.85)	1.90 (1.21, 2.98)	2.07 (0.93, 4.62)
Obese *vs* normal BMI	3.52 (2.36, 5.24)	3.79 (2.37, 6.05)	2.97 (1.26, 7.01)
RA symptom duration, months	1.06 (1.00, 1.12)	1.06 (1.00, 1.13)	1.06 (0.95, 1.17)
DAS-28	1.05 (0.95, 1.17)	1.00 (0.87, 1.14)	1.19 (0.98, 1.44)
RF positive and/or ACPA positive	0.86 (0.62, 1.20)	0.71 (0.48, 1.05)	1.31 (0.68, 2.50)
Comorbidities			
Diabetes	1.65 (0.80, 3.41)	1.80 (0.68, 4.80)	1.53 (0.49, 4.74)
Hyperlipidaemia	1.69 (1.07, 2.66)	2.17 (1.18, 4.02)	1.49 (0.71, 3.13)
Depression	0.86 (0.50, 1.48)	0.94 (0.52, 1.72)	0.40 (0.07, 2.44)

BMI: overweight, 25.0–29.9 kg/m^2^; obese, ≥30.0 kg/m^2^.

High alcohol intake defined as >10 servings for females and >15 servings for males per week.

## Discussion

This study used data from a large inception cohort of early RA and found that a large proportion of patients have comorbid HTN: 26% at baseline and an additional 24% develop it over a median of 5 years of follow-up. Those with traditional CV risk factors (older age, high BMI, diabetes and hyperlipidaemia) at RA diagnosis were most likely to have HTN at baseline and develop HTN over the course of their RA disease.

According to data from the Canadian Health Measures Survey (CHMS) [17], the prevalence of HTN at RA diagnosis in the CATCH cohort was comparable to that of the general Canadian population. However, a large population-based Canadian study of Ontario insurance claims (87 114 RA patients with a mean age of 57 years and unknown RA symptom duration) reported an HTN prevalence at cohort entry of 42%, significantly higher than age- and sex-matched controls [3]. The discrepancy in HTN prevalence compared with our study may be due to different RA disease duration. Studies from other Western countries of RA patients with a disease duration <1 year, analogous to the CATCH cohort, reported HTN prevalence at the time of RA diagnosis of 18–30%, consistent with our findings [18, 19].

Of those who were non-hypertensive at RA diagnosis, one in four patients in our study developed HTN during a median of 5 years of follow-up. This is higher than the HTN incidence of 18% over a mean of 6 years follow-up reported in a population-based study of RA patients with unknown disease duration or severity from the UK. This study also found a higher incidence in RA patients than non-RA controls (13%) [20]. According to a Canadian administrative database study, the HTN incidence in the general population was 3.7% per year for adults 55–60 years of age [21], which would approximate the cumulative incidence of HTN among RA patients in CATCH. However, direct comparison of our RA population with non-RA subjects was not performed in our study and it remains unclear if RA increases the risk of incident HTN. In a study from the USA, RA patients meeting criteria for HTN were 24% less likely to be diagnosed with HTN compared with non-RA controls [22]. A Canadian study reported that only 14% of hypertensive RA patients seen in rheumatology clinics were started on therapies or referred for HTN management [23]. Therefore, studies relying on diagnostic codes for HTN may underestimate the burden of HTN in RA. The reasons for underdiagnosis and undermanagement of HTN in RA are likely multifactorial and include physician barriers, such as lack of time, resources and expertise [23]. Data from this CATCH study does not include the necessary variables to explore quality of care for the management of HTN in early RA.

Similar to what is reported for the general population, we found that early RA patients with the highest risk for HTN were older, overweight/obese and had diabetes or dyslipidaemia—known contributors to vascular dysfunction that predisposes to HTN. In older women, menopause may also be an important factor in HTN risk, as it can lead to increased adipose tissue and insulin resistance [24]. Also, with increasing age, arterial stiffness and structural changes in arteries contribute to an increasing risk of HTN. These physiological changes may be accelerated due to diet, reduced physical activity and inflammation [25]. In the CATCH cohort, dietary variables were not collected, thus we were unable to assess its impact on HTN. However, rates of being obese in this RA population were higher than what is reported in the Canadian population [26]; other important CV risk factors, smoking and diabetes are more frequent in RA patients [3, 27], whereas the proportion with dyslipidaemia was lower in our study [28]. Ascertainment of dyslipidaemia was possibly less accurate in our cohort; lipid levels were not available and dyslipidaemia is underdiagnosed in RA due to low rates of screening and biologic reasons (i.e. decreases in serum low-density lipoprotein cholesterol during active inflammatory disease) [23, 29]. The role of inflammation in HTN risk and reduced physical function remains unclear. Baseline elevations in acute phase reactants and physical function as captured by the validated HAQ were not found to be significantly associated with HTN in CATCH. Chronic inflammation and long-term functional impairment could have a more significant effect, but these factors in the follow-up period were not assessed in this study. We also found that excess alcohol servings are associated with HTN. This is consistent with other studies that have found dose–response associations between alcohol and BP [30].

We did not find baseline disease activity to be associated with incident HTN. While high RA disease activity is characterized by inflammation, pain and reduced physical activity, factors known to contribute to BP elevations [31], other studies did not find significant associations between DAS28 scores and HTN [1, 4]. Therefore, prolonged uncontrolled disease rather than the degree of disease activity at presentation may contribute to the risk of HTN. Similarly, glucocorticoids and COX-2 inhibitors are known risk factors for HTN in RA and other conditions [11], but we did not find that their use at the time of diagnosis was associated with HTN. It is possible that these drugs were avoided in patients with a prior diagnosis of HTN or other comorbidities associated with HTN. The exposure time to DMARDs at study enrolment was low, thus their impact on HTN risk may only be seen when considering long-term exposure.

As in the general population, in our cohort, males had higher prevalent and incident HTN compared with females. Males were also older, had lower education levels, had a higher frequency of cigarette smoking and excess alcohol intake and were more likely overweight/obese and to have a diagnosis of diabetes and dyslipidaemia at baseline, factors known to increase the risk of HTN in the general population. The risk for HTN in overweight/obese and older subjects was more pronounced in females than males, similar to what is reported in the general population [32]; however, it was not statistically significant. The impact of other factors on HTN were similar for males and females. This is in contrast to reports from the general population where smoking, diabetes and dyslipidaemia have a larger impact on HTN in males [32]. Our study may be underpowered to detect sex differences in risk factors for HTN in early RA. Sex differences and the impact of alcohol consumption on HTN in early RA also remain unclear. The risk of prevalent HTN was higher for females with high alcohol consumption (>10 drinks) than males (>14 drinks) at enrolment, but the reverse was found for incident HTN. In a meta-analysis of studies investigating the impact of alcohol use on HTN in the general population, moderate alcohol consumption had a high risk of HTN in males only, but excess consumption had a high risk for both sexes [33]. It is possible that after RA diagnosis, females are more likely than males to decrease their alcohol consumption, reducing their risk of incident HTN. Longitudinal studies are needed to better understand the impact of various risk factors on incident HTN.

This study has several strengths and is one of the largest studies investigating incident hypertension in ERA. The study captured longitudinal BP measurements and explored a wide range of baseline variables as potential risk factors for HTN. Study limitations included the use of self-report to define comorbidities, data on other behaviours that can affect HTN risk (diet, exercise and sleep quality) were not collected, methods for obtaining BP were not standardized across centres, the duration and date of diagnosis of HTN and other comorbidities was not systematically available before 2017 and the impact of time-varying variables over the follow-up period was not assessed. The CATCH cohort includes both academic and community healthcare centres, but it may not be completely representative of the general RA population, in particular marginalized groups that are less likely to participate in research.

## Conclusions

In Canadian early RA patients, HTN prevalence at RA onset appears to be similar to that of the general population. After RA diagnosis, patients have a high risk of developing new HTN. Characteristics associated with HTN in RA patients (older age, diabetes, high BMI, dyslipidaemia and excessive alcohol intake) were also consistent with those of the general population. Given the high frequency of CV risk factors in RA, patients should be routinely screened and modifiable factors managed. Future research should explore risk factors for HTN beyond RA diagnosis, as factors contributing to the risk of HTN and CV events may change as RA disease progresses.

## Supplementary Material

rkae066_Supplementary_Data

## Data Availability

The data underlying this article will be shared upon reasonable request to the corresponding author with permission from CATCH.

## References

[1] Luque Ramos A , Redeker I, Hoffmann F et al Comorbidities in patients with rheumatoid arthritis and their association with patient-reported outcomes: results of claims data linked to questionnaire survey. J Rheumatol 2019;46:564–71. 30647170 10.3899/jrheum.180668

[2] Hitchon CA , Boire G, Haraoui B et al Self-reported comorbidity is common in early inflammatory arthritis and associated with poorer function and worse arthritis disease outcomes: results from the Canadian Early Arthritis Cohort. Rheumatology (Oxford) 2016;55:1751–62. 27330161 10.1093/rheumatology/kew061

[3] Widdifield J , Paterson JM, Huang A, Bernatsky S. Causes of death in rheumatoid arthritis: how do they compare to the general population? Arthritis Care Res 2018;70:1748–55. 10.1002/acr.23548 29512334

[4] Panoulas VF , Metsios GS, Pace AV et al Hypertension in rheumatoid arthritis. Rheumatology (Oxford) 2008;47:1286–98. 18467370 10.1093/rheumatology/ken159

[5] Baghdadi LR , Woodman RJ, Shanahan EM, Mangoni AA. The impact of traditional cardiovascular risk factors on cardiovascular outcomes in patients with rheumatoid arthritis: a systematic review and meta-analysis. PLoS One 2015;10:e0117952. 25689371 10.1371/journal.pone.0117952 PMC4331556

[6] Barra LJ , Pope JE, Hitchon C et al The effect of rheumatoid arthritis-associated autoantibodies on the incidence of cardiovascular events in a large inception cohort of early inflammatory arthritis. Rheumatology (Oxford) 2017;56:768–76. 28073956 10.1093/rheumatology/kew474

[7] Avina-Zubieta JA , Thomas J, Sadatsafavi M, Lehman AJ, Lacaille D. Risk of incident cardiovascular events in patients with rheumatoid arthritis: a meta-analysis of observational studies. Ann Rheum Dis 2012;71:1524–9. 22425941 10.1136/annrheumdis-2011-200726

[8] Aviña-Zubieta JA , Choi HK, Sadatsafavi M et al Risk of cardiovascular mortality in patients with rheumatoid arthritis: a meta-analysis of observational studies. Arthritis Rheum 2008;59:1690–7. 19035419 10.1002/art.24092

[9] Widdifield J , Bernatsky S, Paterson JM et al Trends in excess mortality among patients with rheumatoid arthritis in Ontario, Canada. Arthritis Care Res 2015;67:1047–53. 10.1002/acr.22553 25623141

[10] Kremers HM , Crowson CS, Therneau TM, Roger VL, Gabriel SE. High ten-year risk of cardiovascular disease in newly diagnosed rheumatoid arthritis patients: a population-based cohort study. Arthritis Rheum 2008;58:2268–74. 18668561 10.1002/art.23650 PMC2929699

[11] Hadwen B , Stranges S, Barra L. Risk factors for hypertension in rheumatoid arthritis patients-a systematic review. Autoimmun Rev 2021;20:102786. 33609791 10.1016/j.autrev.2021.102786

[12] Crowson CS , Rollefstad S, Ikdahl E et al Impact of risk factors associated with cardiovascular outcomes in patients with rheumatoid arthritis. Ann Rheum Dis 2018;77:48–54. 28877868 10.1136/annrheumdis-2017-211735 PMC5726925

[13] Bykerk VP , Jamal S, Boire G et al The Canadian Early Arthritis Cohort (CATCH): patients with new-onset synovitis meeting the 2010 ACR/EULAR classification criteria but not the 1987 ACR classification criteria present with less severe disease activity. J Rheumatol 2012;39:2071–80. 22896026 10.3899/jrheum.120029

[14] Hypertension Canada. Diagnosis and assessment. https://guidelines.hypertension.ca/diagnosis-assessment/ (May 2023, date last accessed).

[15] Butt P , Beirness D, Gliksman L, Paradis C, Stockwell T. Alcohol and health in Canada: a summary of evidence and guidelines for low risk drinking. Ottawa: Canadian Centre on Substance Abuse, 2011.

[16] Weir CB , Jan A. BMI classification percentile and cut off points. Treasure Island, FL: StatPearls, 2021. 31082114

[17] DeGuire J , Clarke J, Rouleau K, Roy J, Bushnik T. Blood pressure and hypertension. Health Rep 2019;30:14–21. 30785635

[18] Innala L , Sjöberg C, Möller B et al Co-morbidity in patients with early rheumatoid arthritis – inflammation matters. Arthritis Res Ther 2016;18:33. 26818851 10.1186/s13075-016-0928-y PMC4730785

[19] Gherghe AM , Dougados M, Combe B et al Cardiovascular and selected comorbidities in early arthritis and early spondyloarthritis, a comparative study: results from the ESPOIR and DESIR cohorts. RMD Open 2015;1:e000128. 26535145 10.1136/rmdopen-2015-000128 PMC4623372

[20] Jafri K , Bartels CM, Shin D, Gelfand JM, Ogdie A. Incidence and management of cardiovascular risk factors in psoriatic arthritis and rheumatoid arthritis: a population-based study. Arthritis Care Res 2017;69:51–7. 10.1002/acr.23094 PMC5191972 27696731

[21] Robitaille C , Dai S, Waters C et al Diagnosed hypertension in Canada: incidence, prevalence and associated mortality. CMAJ 2012;184:E49–56. 22105752 10.1503/cmaj.101863 PMC3255225

[22] Bartels CM , Johnson H, Voelker K et al Impact of rheumatoid arthritis on receiving a diagnosis of hypertension among patients with regular primary care. Arthritis Care Res 2014;66:1281–8. 10.1002/acr.22302 PMC4269481 24585741

[23] Ladak K , Hashim J, Clifford-Rashotte M et al Cardiovascular risk management in rheumatoid arthritis: a large gap to close. Musculoskelet Care 2018;16:152–7. 10.1002/msc.1196 28417529

[24] Hallajzadeh J , Khoramdad M, Izadi N et al Metabolic syndrome and its components in premenopausal and postmenopausal women: a comprehensive systematic review and meta-analysis on observational studies. Menopause 2018;25:1155–64. 29787477 10.1097/GME.0000000000001136

[25] Clayton ZS , Craighead DH, Darvish S et al Promoting healthy cardiovascular aging: emerging topics. J Cardiovasc Aging 2022;2:43. 36337728 10.20517/jca.2022.27 PMC9632540

[26] Bhole VM , Choi HK, Burns LC et al Differences in body mass index among individuals with PsA, psoriasis, RA and the general population. Rheumatology (Oxford) 2012;51:552–6. 22120603 10.1093/rheumatology/ker349

[27] Manuel DG , Wilton AS, Bennett C et al Health reports: smoking patterns based on birth-cohort-specific histories from 1965 to 2013, with projections to 2041. Stat Canada 2020;31:16–31. 10.25318/82-003-x202001100002-eng 33205939

[28] Joffres M , Shields M, Tremblay MS, Connor Gorber S. Dyslipidemia prevalence, treatment, control, and awareness in the Canadian Health Measures Survey. Can J Public Health 2013;104:e252–7. 23823891 10.17269/cjph.104.3783 PMC6974261

[29] Venetsanopoulou AI , Pelechas E, Voulgari PV, Drosos AA. The lipid paradox in rheumatoid arthritis: the dark horse of the augmented cardiovascular risk. Rheumatol Int 2020;40:1181–91. 32524301 10.1007/s00296-020-04616-2

[30] Roerecke M , Kaczorowski J, Tobe SW et al The effect of a reduction in alcohol consumption on blood pressure: a systematic review and meta-analysis. Lancet Public Health 2017;2:e108–20. 29253389 10.1016/S2468-2667(17)30003-8 PMC6118407

[31] Petrie JR , Guzik TJ, Touyz RM. Diabetes, hypertension, and cardiovascular disease: clinical insights and vascular mechanisms. Can J Cardiol 2018;34:575–84. 29459239 10.1016/j.cjca.2017.12.005 PMC5953551

[32] Gerdts E , Sudano I, Brouwers S et al Sex differences in arterial hypertension. Eur Heart J 2022;43:4777–88. 36136303 10.1093/eurheartj/ehac470 PMC9726450

[33] Roerecke M , Tobe SW, Kaczorowski J et al Sex-specific associations between alcohol consumption and incidence of hypertension: a systematic review and meta-analysis of cohort studies. J Am Heart Assoc 2018;7:e008202. 29950485 10.1161/JAHA.117.008202 PMC6064910

