# Supplementary material for: Risk factors for prevalent and incident hypertension in rheumatoid arthritis: data from the Canadian Early Arthritis Cohort
Source: Rheumatol Adv Pract. 2024 May 22;8(2):rkae066. doi: 10.1093/rap/rkae066 (PMC11183658; doi:10.1093/rap/rkae066)
Supplement: rkae066_Supplementary_Data [file rkae066_supplementary_data.docx]

**Supplementary Material**

**Supplementary Data S1. CATCH investigators.**

CATCH investigators are: Pooneh Akhavan, Louis Bessette, Gilles Boire, Vivian Bykerk, Ines Colmegna, Sabrina Fallavollita, Derek Haaland, Boulos Haraoui, Glen Hazlewood, Carol Hitchon, Shahin Jamal, Raman Joshi, Ed Keystone, Bindee Kuriya, Peter Panopalis, Janet Pope, Carter Thorne, Edith Villeneuve, Michel Zummer.


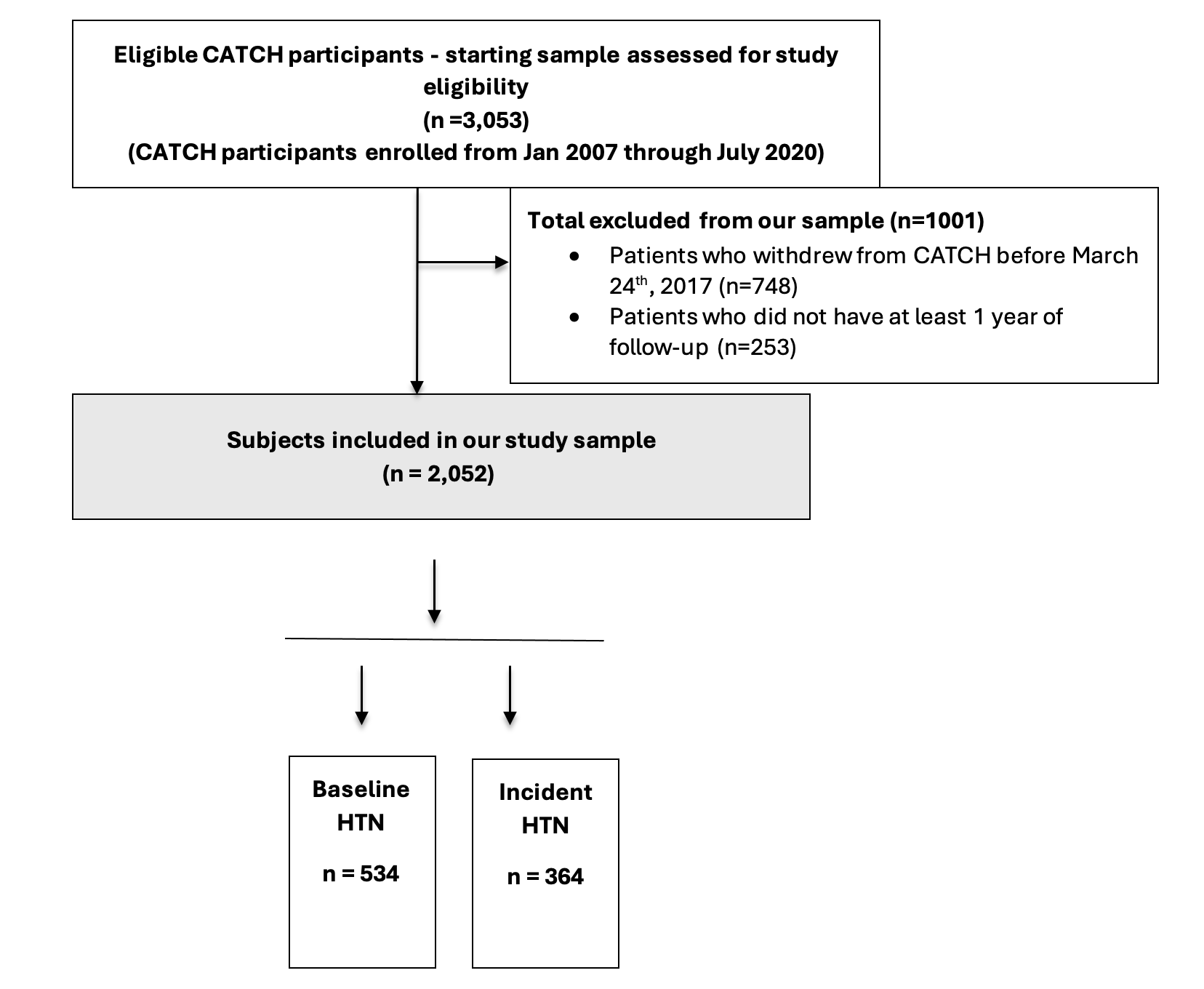


**Supplementary Figure S1. Cohort Flow-Diagram**
